# Self-healing photoluminescent polymers with photosensitive behavior for information storage and multiple-level dynamic encryption†

**DOI:** 10.1039/d4sc02733g

**Published:** 2024-07-19

**Authors:** Di Zhao, Xianglong Li, Qianrui Li, Chunmei Yue, Yige Wang, Huanrong Li

**Affiliations:** a School of Chemical Engineering and Technology, Hebei University of Technology GuangRong Dao 8, Hongqiao District Tianjin 300130 P. R. China lihuanrong@hebut.edu.cn

## Abstract

Photo-responsive luminescent materials capable of responding to light stimuli are crucial in the realm of sophisticated encryption, anti-counterfeiting, and optical data storage. Yet, the development of such materials that also feature self-healing capabilities, swift reaction times, light weight, fatigue resistance, dynamic display abilities, and enhanced security measures is exceedingly rare and presents considerable challenges. Herein, a novel family of self-healing and photo-stimuli-responsive photoluminescent polymers are reported, which is achieved by interlinking terpyridine- and spiropyran-functionalized polymers through N–Ln coordination bonds and hydrogen bonding among the polymer chains. The resulting polymers exhibit good processability, superior tensile strength, fast self-healing ability, and photo-stimuli-responsive performance. The photo-stimuli-responsive properties include unique color shifts (colorless and purple) and light-controlled time-dependent fluorescence modulation (green-, red-, and yellow-emission), which stem from fine-tuning the isomerization of spiropyran and leveraging the fluorescence resonance energy transfer (FRET) from Ln–Tpy donors to spiropyran acceptors, respectively. Besides, these polymers have been successfully applied in dynamic multi-level information encryption applications. We are convinced that these smart materials, crafted through our innovative approach, hold vast potential for applications in information storage, cutting-edge anti-counterfeiting encryption, UV-sensing, and light-writing technologies, marking a novel strategy in the design of photosensitive luminescent smart materials.

## Abstract 2

A family of light-responsive photoluminescent materials with self-healing properties were obtained and applied in dynamic multi-level information encryption applications.

## Graphical abstract

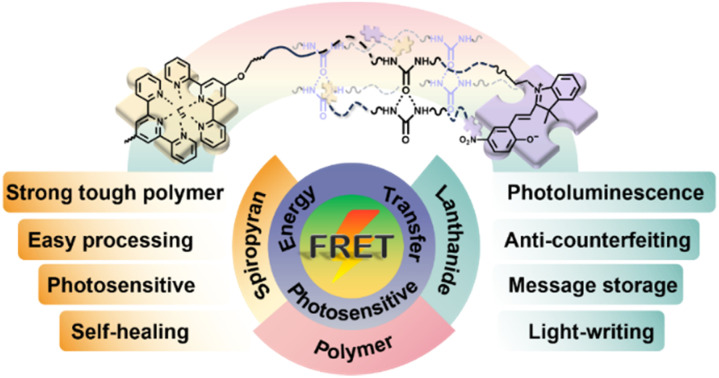

## 1. Introduction

In the swiftly evolving realm of optical information science, there's a pressing demand for innovative information storage and multiple-level encryption platforms that boast high-density storage capabilities, dynamic display features, and cutting-edge security measures.^1,2^ Yet, the development of such sophisticated systems presents significant challenges. So far, one of the effective methods to achieve the above requirements is to use stimulus-responsive photoluminescent (PL) lanthanide-based materials that can quickly switch between different states for information encryption systems and optical data recording.^3,4^ Regarding light-emitting sources, lanthanide complexes are recognized for their superior capabilities in creating luminous materials, attributed to their exceptional photoluminescence characteristics, including large Stokes shifts, narrow emission bands, elevated photoluminescence efficiency, prolonged lifetimes, and the ability to produce multicolor emission.^5,6^

However, the fluorochromic and chromogenic properties in most lanthanide-based studies are mainly obtained *via* the alternating use of different chemical substances (such as ions, acid/alkali, or water) due to the chemical responsiveness feature of these materials.^7,8^ These systems are prone to suffer from the residue/accumulation of chemicals, which would largely reduce the cyclability, durability, reliability, and sensitivity. Light radiation is a green stimulus due to its non-invasiveness and nondestructive nature, submicron- or micron-sized focusing area, controllable energy, time and wavelength, remote control, and precisely controlled direction.^9^ Currently, many elegant photoswitching materials have been reported, in which the implementation of photoswitching ability relies on certain special molecules with chemical structure conversion capacities triggered by light, such as diarylethene (DAE),^10^ spiropyran (SP),^11,12^ azobenzene (AZO),^13^ phosphomolybdic acid hydrate (PMA),^14^*etc.* Among them, spiropyran (SP) has been widely exploited and used in multiple-level encryption fields because it can transform from a non-luminescent closed-ring structure to a luminescent open-ring merocyanine (MC) isomer on UV light treatment and can restore its initial state on visible-light treatment.^15,16^ For example, Tang and his colleagues reported a photosensitive material based on naphthalene and spiropyran, which can be applied to a three-level information encryption platform by artificial design.^17^ Cui's team reported a visible-light-driven photosensitive fluorescent material based on functionalized spiropyran and applied it to the field of dual-level information anti-counterfeiting encryption.^18^ Introducing spiropyran units into the lanthanide-based photoluminescent materials will inevitably combine the advantages of both into one system.

Within a diverse array of sophisticated lanthanide-based materials, including metal–organic frameworks (MOFs),^19^ hydrogen-bonded organic frameworks (HOFs),^20,21^ conventional solid powders,^22^ and hydrogels,^23^ polymer matrices stand out for their ease of processing, robust mechanical characteristics, and versatile functionalities.^24^ Despite these advantages, conventional polymers tend to suffer from damage and breakage due to use, highlighting the essential need to enhance these materials with self-healing properties for sustained durability and performance.^25^ Fortunately, the Ln–ligand coordination bonds and hydrogen bonds within the polymer chains provide the possibility to endow the lanthanide-based polymer with self-healing ability owing to their dynamic nature, which can autonomously repair physical damage, restore the original functions, extend service life, and reduce waste and maintenance costs.^26–28^ We envision that this multifunctional intelligent material, which combines color changes (absorption), fluorescence variations (emission), processing performance, mechanical properties, and self-healing properties, will inevitably generate new possibilities. However, no such studies have been reported yet, to the best of our knowledge.

In this work, we develop a family of advanced, multifunctional intelligent polymers, combining exceptional mechanical properties, swift light-responsiveness, efficient processability, recyclability, and fast self-healing behavior, by intertwining terpyridine (Tpy)- and spiropyran (SP)-modified polyurethane chains through Tpy–Ln coordination and hydrogen bonds within the polymer framework. Spiropyran stands out as a key photosensitive element in light-activated systems, capable of switching between its closed-ring (SP) and open-ring merocyanine (MC) forms upon exposure to alternating UV and visible light, making it an integral photosensitive component of the polymer structure. The lanthanide complexes retain the exceptional luminescence qualities of the respective Ln^3+^ ions while also ensuring spectral overlap with the MC-state of spiropyran to modulate the photoluminescence of the polymers by using photo-stimuli. Leveraging the synergistic properties of Tpy–Ln and spiropyran, the well-known Förster resonance energy transfer (FRET) process from the Tpy–Ln donor to the spiropyran acceptor was induced by UV irradiation, and swift fluorescence color modulation (including green to red, yellow to red and green to yellow) along with distinct color transformation (colorless to purple) ability was achieved. The color and luminescence properties of the resultant deep purple polymer could be restored upon visible light irradiation. In addition to comprehensively exploring the superior performance of our polymers, these polymers have been successfully employed in dynamic and controllable applications of multi-level information encryption. We are confident that these advanced, multifunctional intelligent polymers will inevitably pave the way in the fields of sophisticated information encryption and multi-level anti-counterfeiting technologies.^29–31^

## 2. Results and discussion

### 2.1 Materials design and synthesis

**Fig. 1:**
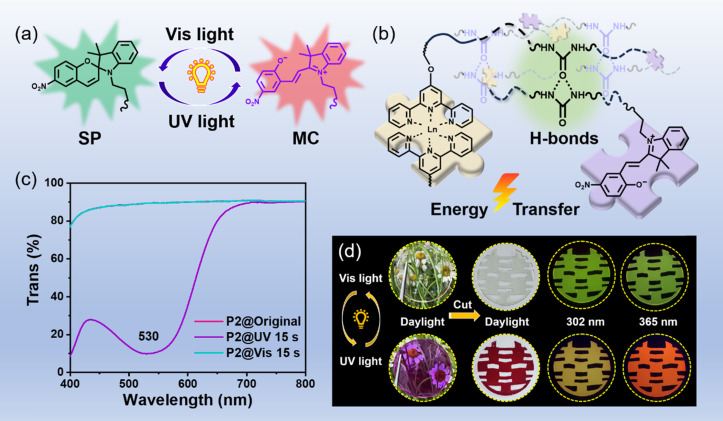
(a) Chemical structure conversion illustration of spiropyran from a closed-ring structure (SP) to an open-ring merocyanine (MC) isomer. (b) A simple schematic diagram of the chemical structure of samples P2 and P3 (for P2, Ln = Tb^3+^; for P3, Ln = Eu^3+^; please refer to Fig. S1† for the specific chemical structure). (c) Transmission spectra of photosensitive behavior of the representative sample P2. (d) Processing ability and photo-controlled luminescence modulation capability of the representative sample P2.

The key design concept in the construction of our photosensitive materials is to introduce both the photo-stimuli-responsive spiropyran section and the photoluminescent Tpy–Ln segment into the tough self-healing polymer matrix. According to the above-mentioned design concept, a series of photo-controlled photoluminescent polymers and comparison samples were successfully synthesized, and the feed ratio and synthesis route are shown in Table S1 and Fig. S1,† respectively. The Tb-containing photosensitive polymer (marked as the P2 sample) was chosen as the representative sample for further research, and its successful synthesis was proved *via* Fourier transform infrared (FT-IR) (Fig. S2†), rheological testing (Fig. S3†) and gel permeation chromatography (GPC) characterization (Fig. S4†). Moreover, the resulting P2 sample shows low *T*_g_ (−53 °C, see Fig. S5†), numerous hydrogen bonds (Fig. S6†), and typical amorphous structures (Fig. S7†). The obtained photosensitive polymers P2 containing a ring-closed spiropyran (SP) form are colorless and transparent (Fig. 1), with an 89.1% average transmittance in the visible range of 400–800 nm (Fig. S8†). After being exposed to 365 nm UV light for only 15 s, spiropyran components can transform into ring-opened merocyanine (MC), and the photosensitive polymers can be highly purple-colored (Fig. 1).^32^ Meanwhile, the comparison samples (P0, P4, and P5) without spiropyran components cannot achieve the above-mentioned photochromic performance (Fig. S9†), and can only consistently exhibit a transmittance of ∼89% in the visible light range (Fig. S9†). In addition, the obtained target photosensitive polymer (represented by P1 and P2) and comparison samples (represented by P0 and P4) all present a relatively high decomposition temperature of 223 °C (Fig. S10†).

### 2.2 Mechanical properties and self-healing capacity

**Fig. 2:**
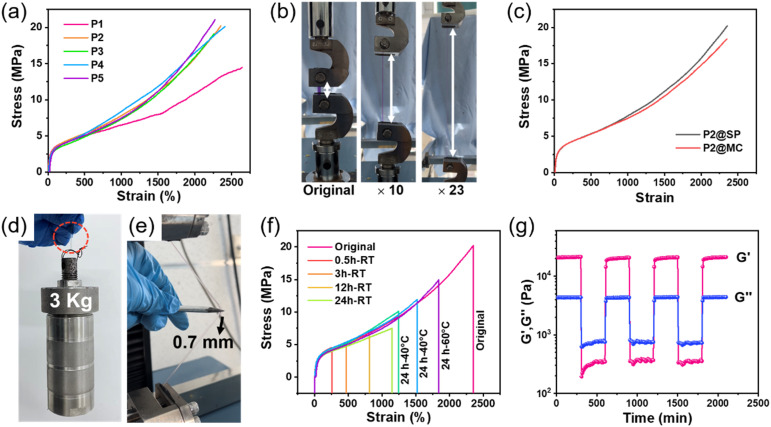
(a) Stress–strain curves of P1–P5. (b) Stretching images of the P2 sample. (c) Stress–strain curves of P2 with SP and MC. (d) Load bearing capacity and (e) puncture resistance test of the sample P2 polymer strip. (f) Stress–strain curves of fractured photosensitive P2 samples self-healed for different times at room temperature (RT) and for 24 h at different temperatures (stretch speed: 50 mm min^−1^). (g) Alternate step strain rheological measurement of the storage modulus (*G*′) and loss modulus (*G*′′) of P2.

Firstly, tensile measurements were conducted to evaluate the mechanical properties of the obtained photosensitive polymers, and the results are summarized in Fig. 2a and Table S2.† We chose P2 as the representative sample for the following illustration and research. The prepared P2 exhibits high tensile strength and superior stretchability due to its reasonable molecular design, which could be stretched to 23× its original length without breaking (Fig. 2b), accompanied by a high tensile strength of 20.19 ± 0.86 MPa, a high Young's modulus of 13.63 ± 0.40 MPa and an ultra-high toughness of 229.79 ± 12.65 MJ m^−3^ (Fig. 2c). The mechanical properties of our polymers with a stretchability of 2354 ± 48% and a tensile strength of 20.19 ± 0.86 MPa are more advantageous compared with that of the previously reported photosensitive materials,^14,33–35^ undoubtedly endowing our material with the ability to maintain safety and durability in harsh and complex usage environments.^36^ Moreover, due to the absence of the toughening effect of Tpy–Ln coordination bonds, the photosensitive sample P1 showed a slight decrease in tensile stress of 14.48 ± 0.39 MPa, and a higher strain of 2643 ± 72% (Fig. 2a and Table S2†), while the mechanical properties of P3, P4, and P5 samples are similar to those of P2, indicating that the presence of a small amount of spiropyran (<0.2%) and the influence of lanthanide ion types on the material's mechanical properties can be ignored (Fig. 2a and Table S2†).

After treating photosensitive polymer P2 with 365 nm ultraviolet or visible light, there was no significant change in its mechanical properties (Fig. 2c). This observation indicates that the luminescence switching process does not affect the mechanical behavior of the metal–polymer, *i.e.*, its open and closed isomerization does not affect the polymer network.^37^ The outstanding mechanical properties can also be proved *via* a load-bearing measurement and puncture resistance experiment. As shown in Fig. 2d, a 3 kg reaction kettle bucket could be lifted by a 92 mg P2 sample strip, whose weight is more than 32 600 times that of the P2 strip. Then, we fixed the strain of a 0.2 mm thick photosensitive sample at 500%, and the sample was not punctured by a 0.7 mm sharp steel needle under manual sideway stretching (Fig. 2e). Also, the synthesized photosensitive polymers exhibit fast elastic restorability, indicating fatigue resistance and reliability of the materials during repeated deformation. As shown in Fig. S11,† after sideway stretching and release under a fixed deformation of 500%, the stretched P2 recovered instantly like a piece of rubber. Similar to typical elastomers reported previously,^38,39^ the prepared polymers also exhibit a certain degree of viscoelasticity, which can be demonstrated through cyclic tensile testing. As shown in Fig. S12,† significant hysteresis was obtained, during the continuous loading and unloading tensile tests, indicating the presence of energy dissipation (the specific values are summarized in Table S3†), which is ascribed to the dissociation/recombination of dynamic crosslinking bonds that cannot be fully restored to their original state at a single cyclic time scale.^40,41^

Next, the self-healing performance of the photosensitive polymers was studied. As shown in Fig. S13 and Video S1,† the fractured P2 sample can withstand a strain of 150% after self-healing for only 2 min. To further determine the self-healing ability, P2 samples were bisected and allowed to self-heal at room temperature (RT) for different times, and then stretched until failure again. The stress, stain and toughness of the samples were restored to 7.49 ± 0.53 MPa, 1144 ± 31% and 59.33 ± 0.30 MJ m^−3^, respectively, after self-healing at RT for 24 h, without any external stimulus (Fig. 2f and Table S4†). Moreover, the healing ability of P2 can be improved by heating. The stress, strain and toughness of P2 recover to 1842 ± 33, 14.92 ± 0.43 MPa and 140.25 ± 10.3 MJ m^−3^, respectively, after self-healing at 60 °C for 24 h (Fig. 2f and Table S4†). The self-healing properties of the prepared polymers originate from the dynamic nature of hydrogen bonds and Tpy–Tb coordination interactions, which can be demonstrated by alternate step strain rheological measurement.^42^ As shown in Fig. 2g, the elastomer can transfer from the solid state (*G*′ > *G*′′) at 0.1% shear strain to the liquid state (*G*′ < *G*′′) at 100% strain due to the breaking of the dynamic networks. After the strain recovers to 0.1%, the fractured dynamic bonds are reconstructed and the polymer network is restored to the solid state again (*G*′ > *G*′′) and the responses of our polymer can rapidly alternate between the above two states. The non-crystallized loose structure (Fig. S7†) and low *T*_g_ (−53 °C, see Fig. S5†) also provide a driving force for the diffusion and movement of polymer chains, thereby promoting the self-healing behavior of the material.^43^

As a comparison, our photosensitive material P1 has a higher self-healing efficiency than that of P2 at room temperature for a short self-healing time (0.5, and 3 h) (Fig. S14 and Tables S4, S5†). With the increase in self-healing time (12, and 24 h), the self-healing efficiency of P2 exceeds that of P1. In addition, the self-healing efficiency of P2 will also be higher than that of P1 after increasing the temperature (Tables S4 and S5†). We speculate that the above phenomenon is due to the absence of Tpy–Ln coordination bonds in P1, which leads to higher diffusion of polymer chains at room temperature and facilitates the self-healing of damaged materials. As the temperature increases, polymer chains undergo thermally induced movement and diffusion, and the dynamic properties of Tpy–Ln coordination bonds drive the self-healing performance of the polymer. Furthermore, the obtained photosensitive polymers can be dissolved in DCM solvents, and then be re-cast and dried to obtain recycled samples. The mechanical properties and photosensitivity of the recovered samples are maintained, indicating that our materials have good reusability and recycling ability (Fig. S15†).

### 2.3 Light-control luminescence performance

**Fig. 3:**
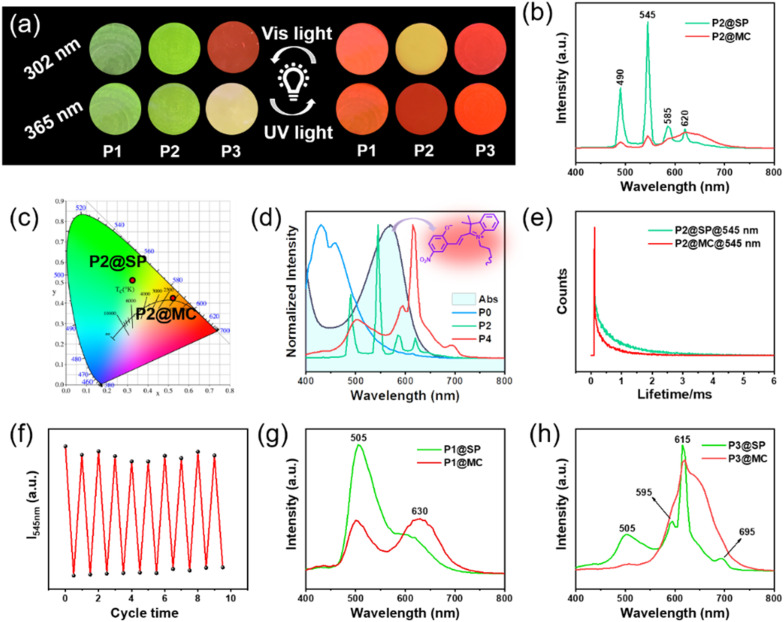
(a) Photoluminescence (PL) digital images of photosensitive polymers P1, P2, and P3 after 15 s of 365 nm UV light or 15 s of visible light treatment. (b) PL spectra of materials P2 excited at 325 nm at both the SP-state and MC-state and (c) their corresponding emission colors in the CIE 1931 diagrams. (d) Overlaps between the emission spectra of P0, P2, and P3 (donor) and the absorption curve of MC (acceptor). (e) Decay curves of P2 at both the SP-state and MC-state monitored at 545 nm. (f) PL intensity at 545 nm of representative sample P2 during cyclic exposure to UV/Vis radiation. PL spectra of (g) P1 and (h) P3, excited at 325 nm at both the SP-state and MC-state.

As shown in Fig. 3a, along with adjusting the feed ratio of the raw materials, fascinating luminescence color changes were obtained after 15 s of 365 nm UV radiation, including green-emission to red-emission (P1 under 302 and 365 nm; P2 under 365 nm), green-emission to yellow-emission (P2 under 302 nm), yellow-emission to red-emission (P3 under 365 nm), and red-emission retention (P3 under 302 nm). The color and luminescence properties of the resultant deep purple polymer could be restored to the initial state upon visible light irradiation for 15 s (Fig. 1). Next, the Tb^3+^-containing sample (marked as P2) was chosen as the representative example to further study the light-control photosensitive performance of as-prepared materials. In its original SP-state, the P2 sample is transparent and colorless under daylight and the spiropyran units show absorption below 400 nm (Fig. 1c). There is no spectral overlapping between the absorption of spiropyran units and the emission of the Tpy–Tb component. Therefore, no FRET process occurred, and the photosensitive polymers exhibit the characteristic green emission color of Tpy–Tb under UV light (Fig. 3a–c).^44^ The excitation spectrum of P2 shows a broadband excitation spectrum in the range of 225 to 425 nm centered at 325 nm (Fig. S16†), and when excited at 325 nm its emission spectrum shows emission peaks centered at 490, 545, 585, and 620 nm, which are attributed to ^5^D_4_ → ^7^F_*J*_ (*J* = 6, 5, 4, 3) transitions, respectively, in which the peak at 545 nm generated by ^5^D_4_ → ^7^F_5_ is responsible for the characteristic green-emission of Tb^3+^ (Fig. 3b and c).^5^

**Fig. 4:**
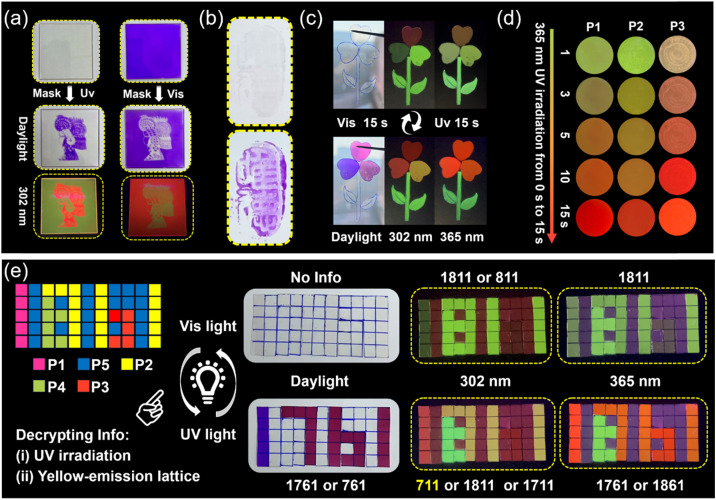
(a) Light-writing properties of the obtained photosensitive polymer (8 cm × 8 cm). (b) The application of the prepared materials in invisible ink (the text means “Only morality”). (c) Patterns of anti-counterfeiting constructed by using P1, P2, P3, and P4 polymer samples. (d) Time-dependent photosensitive fluorescence color change process of P1, P2, and P3 induced by 365 nm UV light irradiation. (e) Information storage and multiple-level anti-counterfeiting encryption platforms prepared by using P1, P2, P3, P4, and P5 which can be decoded/encoded *via* UV/vis light treatment, respectively.

After exposure to 365 nm UV light for 15 s, the spiropyran unit within the luminescent polymers switched from ring-closed SP to the ring-open merocyanine form (MC), and the material rapidly transformed into a deep purple color (see Video S2† and Fig. 1), which generates new absorption bands in the range of 445–650 nm. This 445–650 nm absorption range overlaps with the luminescence emission range of the Tpy–Tb components, and therefore efficient photoinduced FRET from Tb^3+^ to MC in the polymers arose (Fig. 3d). Therefore, the luminescence intensity of Tpy–Tb in the material was quenched by 90.13% with concomitant decreases in the ^5^D_4_ decay times from 0.62 to 0.36 ms (Fig. 3e). According to the previously reported method, the FRET efficiency in the polymer was calculated to be 42%.^45,46^ Meanwhile, due to the addition of red-emission (characteristic broadband centered at 625 nm) originating from MC, the luminescence changed from green to a clear yellow-emission under 302 nm UV light, and luminescence transformed from green-emission to bright-red-emission under 365 nm UV light (Fig. 4a). Moreover, the color and luminescence properties of the resultant deep purple polymer could be restored to the initial state upon visible light irradiation for only 15 s, arising from the regeneration of SP (Video S2†).

Notably, the reversible and prominent luminescence photosensitive behavior of P2 could be steadily switched by alternating UV and visible-light irradiation, and the minimum and maximum PL intensities at 545 nm do not present obvious decay (Fig. 4f). The cyclic characteristics can be reproduced at least ten times without fatigue, indicating that the obtained photosensitive polymer has excellent stability and fatigue resistance as a potential dynamic fluorescent material. As for P1 and P3, their PL spectra and CIE diagrams also correspond to their digital photos (Fig. 4a, g, h and S17†).

### 2.4 Light-writing and information encryption properties

Non-contact UV light (365 nm) and visible light can be used as an ideal “pen” to further explore the real-time light-writing display of transient information. Using Chinese paper cuttings as a mask, and then using ultraviolet (see Fig. 4a left) or visible light (see Fig. 4a right) as a pen, we can easily obtain the corresponding portraits of cartoon characters by screen printing, and the cartoon characters can also be identified under 302 nm UV light (Fig. 4a). Also, the prepared materials can be used as invisible ink the obtain photosensitive text, which can only be read after being irradiated with 365 nm UV light (Fig. 4b). In addition, due to the excellent fatigue resistance and cycling characteristics of our polymer's photosensitive performance, dynamic information “H”, “E”, “B”, “U”, and “T” can be easily written onto a single sample film through a write-erase-rewrite process (Fig. S18†).

Usually, traditional luminescent encryption materials can be easily identified and have hidden dangers due to the characteristics of single-level encryption modes.^47,48^ Therefore, it is necessary to develop higher levels of protection for information storage and passing. We believe that the photosensitive materials prepared by us feature broad application prospects in the field of information anti-counterfeiting due to their ability to simultaneously change color (absorption) and show various time-dependent photosensitive fluorescent color changes (emission) in response to external light stimuli. As shown in Fig. 4c, a pattern anti-counterfeiting platform was constructed *via* P1, P2, P3, and P4 (for distribution of various samples, see Fig. S19†). The color change (for first-level encryption) (see Fig. 4c) and time-dependent photosensitive fluorescence under 365 nm UV light (for second-level encryption) can be realized (Fig. 4d), indicating their unique applicability in the field of anti-counterfeiting. Interestingly, different parts of the material can quickly self-heal and bear their own weight after coming into contact with the fracture surface of the cut material, even though there is only a very small contact area of the fracture surface due to the lightweight nature and fast room temperature self-healing ability of the obtained materials (Fig. 4c).

In addition, carefully crafted and well-designed multiple-level information encryption matrices can also be achieved, which calls for two consecutive actions to unveil the encrypted information. These two consecutive actions are (i) irradiating the information matrices at 365 nm for 15 s, and (ii) observing the information formed by the yellow lattice as real information. As shown in Fig. 4e, we set information “711” formed by the yellow lattice as correct information. Initially, there is no information available in the 5 × 11 transparent sample arrays under daylight (for first-level encryption), and information “811” and “1811” can be detected under 302 and 365 nm UV light, respectively (for second-level encryption). Subsequently, after exposure to 365 nm UV light for only 15 s, the information “1671” and “761” can be identified under daylight (for third-level encryption), information “711”, “1711” and “1811” can be detected at 302 nm, “1761”, and “1861” can be detected at 365 nm (for fourth-level encryption). So, a fourth-level information encryption platform was achieved. The sample information arrays can be restored to their original state and encrypted again by exposure to visible light for 15 s. Among the above-mentioned P1, P2 and P3 pixel samples, there is also a time-dependent photosensitive fluorescence behavior (please refer to Fig. 4d), which also plays a role in advanced anti-counterfeiting.

## 3. Conclusion

In summary, a series of visible/UV-light-driven photosensitive photoluminescent polymers with superior mechanical properties and self-healing performance were successfully prepared. On regulating the isomerization of spiropyran components and the FRET between Ln–Tpy donors and spiropyran receptors, distinct color change (absorption switching, colorless to purple) and rapid fluorescence modulation (emission color alteration, including green-emission to red-emission, yellow-emission to red-emission, green-emission to yellow-emission and red-emission retention) was achieved. The obtained polymers also show superior processing performance, superior mechanical properties (stress: 13.63 MPa, strain: 2300%, and toughness: 229.79 MJ m^−3^), and rapid room temperature self-healing performance. Through our study, we have showcased the immense potential of these materials in applications of photo-rewritable patterns and multi-level information encryption technologies. We firmly believe that the straightforward yet versatile strategy employed in this study offers valuable insights, enriching design concepts, and paving the way for the development of multifunctional photosensitive intelligent materials.

## Data availability

The data supporting this article have been included as part of the ESI.†

## Author contributions

H. Li and D. Zhao designed the experiments and drafted the manuscript, D. Zhao performed the experiments. The manuscript was written with the contributions of all authors.

## Conflicts of interest

There are no conflicts to declare.

## Supplementary Material

SC-015-D4SC02733G-s001

SC-015-D4SC02733G-s002

SC-015-D4SC02733G-s003

## References

[cit1] Xie Y., Sun G., Li J., Sun L. (2023). Adv. Funct. Mater..

[cit2] Wang H., Tang J., Deng H., Tian Y., Lin Z., Cui J., Chen J. (2023). J. Mater. Chem. C.

[cit3] Zhao D., Yang J., Tian X., Wei J., Li Q., Wang Y. (2022). Chem. Eng. J..

[cit4] Li Y., Yang Y., Guo X., Chen Y., Wang Z., Hu L., Wu W., Zhu J. (2023). ACS Appl. Nano Mater..

[cit5] Singh A. K. (2022). Coord. Chem. Rev..

[cit6] Singh P., Kachhap S., Singh P., Singh S. (2022). Coord. Chem. Rev..

[cit7] Zhao D., Yue C., Li Q., Guo L., Yang J., Li H. (2023). ACS Appl. Polym. Mater..

[cit8] Feng P., Yang X., Feng X., Zhao G., Li X., Cao J., Tang Y., Yan C.-H. (2021). ACS Nano.

[cit9] Dong Y., Wu H., Liu J., Zheng S., Liang B., Zhang C., Ling Y., Wu X., Chen J., Yu X. (2024). Adv. Mater..

[cit10] Hou L., Ringström R., Maurer A. B., Abrahamsson M., Andréasson J., Albinsson B. (2022). J. Am. Chem. Soc..

[cit11] Bhattacharyya S., Maity M., Chowdhury A., Saha M. L., Panja S. K., Stang P. J., Mukherjee P. S. (2020). Inorg. Chem..

[cit12] Rad J. K., Balzade Z., Mahdavian A. R. (2022). J. Photochem. Photobiol., C.

[cit13] Li Y., Xue B., Yang J., Jiang J., Liu J., Zhou Y., Zhang J., Wu M., Yuan Y., Zhu Z. (2023). Nat. Chem..

[cit14] Yimyai T., Crespy D., Pena-Francesch A. (2023). Adv. Funct. Mater..

[cit15] Klajn R. (2014). Chem. Soc. Rev..

[cit16] Shiraishi Y., Yomo K., Hirai T. (2023). ACS Phys. Chem. Au.

[cit17] Yang Y., Li A., Yang Y., Wang J., Chen Y., Yang K., Tang B. Z., Li Z. (2023). Angew. Chem..

[cit18] Li X., Wang H., Chen J., Tian Y., Xiang C., Liu W., Zhou Z., Cui J., Chen X. (2023). Adv. Funct. Mater..

[cit19] Xia T., Cao W., Cui Y., Yang Y., Qian G. (2021). Opto-Electron. Adv..

[cit20] Liu X., Ye Y., He X., Niu Q., Chen B., Li Z. (2024). Angew. Chem., Int. Ed..

[cit21] Xu X., Wang J., Yan B. (2021). Adv. Funct. Mater..

[cit22] Cai Y., Yang Y., Liu H., Song N., He H., Wang J. (2022). Inorg. Chem..

[cit23] Martínez-Calvo M., Kotova O., Möbius M. E., Bell A. P., McCabe T., Boland J. J., Gunnlaugsson T. (2015). J. Am. Chem. Soc..

[cit24] Zhao D., Yue C., Li Q., Guo L., Li H. (2023). J. Mater. Chem. C.

[cit25] Wang Y., Shu R., Zhang X. (2023). Angew. Chem..

[cit26] Wei Z. X., Wang H. Q., Li C. H. (2024). J. Polym. Sci..

[cit27] Chen J., Gao Y., Shi L., Yu W., Sun Z., Zhou Y., Liu S., Mao H., Zhang D., Lu T. (2022). Nat. Commun..

[cit28] Cooper C. B., Root S. E., Michalek L., Wu S., Lai J.-C., Khatib M., Oyakhire S. T., Zhao R., Qin J., Bao Z. (2023). Science.

[cit29] Wang X., Xu J., Zhang Y., Wang T., Wang Q., Li S., Yang Z., Zhang X. (2023). Nat. Commun..

[cit30] Chen Y., Mellot G., van Luijk D., Creton C., Sijbesma R. P. (2021). Chem. Soc. Rev..

[cit31] Chen Y., Lee Y. R., Wang W., Fang Y., Lu S., Han J., Chen X., Kim M. H., Yoon J. (2023). Angew. Chem..

[cit32] Alidaei-Sharif H., Babazadeh-Mamaqani M., Roghani-Mamaqani H., Kalajahi M. S. (2023). Eur. Polym. J..

[cit33] Lv X., Yan H., Wang Z., Dong J., Liu C., Zhou Y., Chen H. (2023). Opt. Mater..

[cit34] Deng H., Wang H., Tian Y., Lin Z., Cui J., Chen J. (2023). Mater. Horiz..

[cit35] Li Z., Chen H., Li B., Xie Y., Gong X., Liu X., Li H., Zhao Y. (2019). Advanced Science.

[cit36] Li C. H., Zuo J. L. (2020). Adv. Mater..

[cit37] Yang Y., Li Y., Chen Y., Wang Z., He Z., He J., Zhao H. (2022). ACS Appl. Mater. Interfaces.

[cit38] Xiang X., Zhang L., Sheng D., Yang X., Qi X., Wei S., Dai H. (2024). Adv. Funct. Mater..

[cit39] Wang X., Zhan S., Lu Z., Li J., Yang X., Qiao Y., Men Y., Sun J. (2020). Adv. Mater..

[cit40] Xu J., Wang X., Zhang X., Zhang Y., Yang Z., Li S., Tao L., Wang Q., Wang T. (2023). Chem. Eng. J..

[cit41] Jing T., Heng X., Jingqing T., Haozhe L., Li L., Pingyun L., Xiaode G. (2023). Chem. Eng. J..

[cit42] Zhang G., Ge F., Wang M., Liu Z., Ren Y., Fu K., Wei M., Zhang Q. (2023). Ind. Eng. Chem. Res..

[cit43] Hu P., Zhang Y., Zhou S., Chen T., Wang D., Liu T., Wang Y., Chen J., Wang Z., Xu J. (2023). Chem. Eng. J..

[cit44] Li C., Zhang Y., Hu J., Cheng J., Liu S. (2010). Angew. Chem..

[cit45] Miyata K., Konno Y., Nakanishi T., Kobayashi A., Kato M., Fushimi K., Hasegawa Y. (2013). Angew. Chem., Int. Ed..

[cit46] Feng T., Ye Y., Liu X., Cui H., Li Z., Zhang Y., Liang B., Li H., Chen B. (2020). Angew. Chem..

[cit47] Yue C., Zhao D., Yang J., Li H. (2024). Prog. Org. Coat..

[cit48] Zou S., Bao G., Liu X., Niu Q., Sun C., Li Z., Wang J. (2024). Dyes Pigm..

