# Supplementary material for: Self-healing photoluminescent polymers with photosensitive behavior for information storage and multiple-level dynamic encryption
Source: Chem Sci. 2024 Jul 19;15(33):13306–12. doi: 10.1039/d4sc02733g (PMC11339966; doi:10.1039/d4sc02733g)
Supplement: SC-015-D4SC02733G-s001 [file SC-015-D4SC02733G-s001.pdf]

Electronic Supplementary Material (ESI) for Chemical Science.
This journal is © The Royal Society of Chemistry 2024

Supporting information

Self-healing photoluminescent polymers with photosensitive behavior for information storage and multiple-level dynamic encryption

Di Zhao [a], Xianglong Li [a], Qianrui Li [a], Chunmei Yue [a], Yige Wang [a]*, and Huanrong Li [a]*

[a] School of Chemical Engineering and Technology, Hebei University of Technology, GuangRong Dao 8, Hongqiao District, Tianjin 300130, P. R. China.

Corresponding author: E-mail: lihuanrong@hebut.edu.cn

1. Experimental section

1.1 Materials

Unless otherwise noted, all the reactants and solvents were commercial without further purification. Tolylene-2,4-diisocyanate-terminated polypropylene glycol (PPG-NCO; Mn ≈ 2300), 4′-chloro-2,2′:6′,2′-tripyridine and 5-amino-1-pentanol were purchased from Sigma-Aldrich. 1,6-hexanediamine (HDA) were purchased from Meryer Chemical Reagent. Dibutyltin dilaurate (DBTDL) and 3',3'-Dimethyl-6-nitro-spiro[2H-1-benzopyran-2,2'-indoline]-1'-ethanol (SP-OH) were obtained from Macklin. Dimethyl sulfoxide (DMSO), KOH, methanol (MeOH), trichlormethane (TCM) and dichloromethane (DCM) was purchased from Kemat (Tianjin) Chemical Technology Co., Ltd. DMSO was dried by molecular sieves before use. 5-([2,2';6',2"]-Terpyridine-4'-yloxy)-pentylamine (Tpy-NH_2) was prepared according to the reported literature.

1.2 Synthesis of 5-([2,2';6',2"]-Terpyridine-4'-yloxy)-pentylamine (Tpy-NH_2):

5-aminopentane-1-pentanol (770 mg, 7.47 mmol) was added to a suspension containing KOH (2.00g, 35.6 mmol) and dried DMSO (30 mL), and then the mixture was stirred at 60 °C for 30 min. Then, 4′-chloro-2,2′:6′,2′-tripyridine (2g, 7.47 mmol) was added to the above mixture followed by stirring at 60 °C for 48 h. After that, the mixture was allowed to cool down and poured into 1000mL of deionized water. The

solution was then centrifuged and washed until pH =7. Finally, the product was dried at 60 °C in a vacuum oven for 3 d to obtain yellow solid powder (2g, 83.3%). ^{1}H NMR (500 MHz, $CDCl_3$) δ 8.68 (s, 2H), 8.59 (s, 2H), 7.99 (s, 2H), 7.83 (s, 2H), 7.32 (s, 2H), 4.22 (s, 2H), 2.73 (s, 2H), 1.86 (s, 2H), 1.53 (s, 4H), 1.38 (s, 2H).

1.3 Synthesis of a series of polymers:

Note S1: A series of polymers were synthesized through a similar process by adjusting the feed ratios of the raw materials, (see **Figure S1** and **Table S1**).

Note S2: Our samples are labeled as **P*x*** (*x*=0, 1, 2, 3, 4, 5), where P is the abbreviation for polymer, and the following *x* represent the numbering of different samples.

Synthesis of P0:

PPG-NCO (0.5 mmol, 1.15 g) and DBTDL (10 μL) were dispersed in 10 mL TCM under magnetic stirring at room temperature (RT), and then HDA (0.4 mmol, 46 mg) dissolved in 5 mL DCM was added into the above system, and further stirred at RT for 6 h. Afterward, 2 mL MeOH were added into the above system and further stirred at RT for 1 h at room temperature to quench the reaction. The obtained solution was then cast into the PTFE mold at RT for 12 h and then dried at 50 °C for 24 h to obtain the polymer **P0**. The resulting polymer **P0** was characterized by gel permeation chromatography (GPC) with a molecular weight of Mn= 18115 g/mol, Mw= 45064 g/mol and a polydispersity of 2.488, see **Figure S4a**).

Synthesis of P1:

PPG-NCO (0.5 mmol, 1.15 g) and DBTDL (10 μL) were dispersed in 10 mL TCM under magnetic stirring at room temperature (RT), and then HDA (0.4 mmol, 46 mg) dissolved in 5 mL DCM was added into the above system, and further stirred at RT for 2 h. Afterward, SP-OH (2 mg) dispersed in 1 mL DCM was slowly added into above solution with stirring for 6 hour at room temperature. Afterward, 2 mL MeOH were added into the above system with stirring for 1 h at room temperature (RT) to quench the reaction. The obtained solution was then cast into the PTFE mold at RT for 12 h and then dried at 50 °C for 24 h to obtain the polymer **P1**. The resulting polymer **P1** was characterized by GPC with a molecular weight of Mn= 19392 g/mol, Mw= 46680

g/mol and a polydispersity of 2.407, see **Figure S4b**)

Synthesis of P2 and P3:

PPG-NCO (0.5 mmol, 1.15 g) and DBTDL (10 μL) were dispersed in 10 mL TCM under magnetic stirring at room temperature (RT), and then HDA (0.4 mmol, 46 mg) dissolved in 5 mL DCM was added into the above system, and further stirred at RT for 2 h to prepare the intermediate (noted as **HPU**). Then Tpy-NH_2 (15 mg) dispersed in 3 mL DCM was slowly added into above solution with stirring for 6 h. The obtained material of this step was denoted as **HPU-Tpy**. Afterward, SP-OH (2 mg) dispersed in 1 mL DCM was slowly added into above solution with stirring for 6 hour at room temperature. Afterward, the ethanol solution of $TbCl_3 \cdot 6H_2O$ (0.1 mol L^{-1}, 225 μL) and 2 mL MeOH were added into the above **HPU-Tpy-SP** system with stirring for 1 hours at RT to obtain the final product **P3**. The **P3** solution was then cast into the PTFE mold at room temperature overnight and then dried at 50 °C for 24 h to the constant weight. The resulting polymer **P2** was characterized by GPC with a molecular weight of Mn= 20262 g/mol, Mw= 42708 g/mol and a polydispersity of 2.108, see **Figure S4c**). Keeping all other conditions unchanged, polymer **P3** can be prepared by replacing $TbCl_3 \cdot 6H_2O$ with $EuCl_3 \cdot 6H_2O$.

Synthesis of P4 and P5:

PPG-NCO (0.5 mmol, 1.15 g) and DBTDL (10 μL) were dispersed in 10 mL TCM under magnetic stirring at room temperature (RT), and then HDA (0.4 mmol, 46 mg) dissolved in 5 mL DCM was added into the above system, and further stirred at RT for 2 h to prepare the intermediate (**HPU**). Then Tpy-NH_2 (15 mg) dispersed in 3 mL DCM was slowly added into above solution with stirring for 6 h. The obtained material of this step was denoted as **HPU-Tpy**. Afterward, the ethanol solution of $TbCl_3 \cdot 6H_2O$ (0.1 mol L^{-1}, 225 μL) and 2 mL MeOH were added into the above **HPU-Tpy** system with stirring for 1 hours at RT to obtain the final product **P4**. The **P4** solution was then cast into the PTFE mold at room temperature overnight and then dried at 50 °C for 24 h to the constant weight. The resulting polymer **P4** was characterized by GPC with a molecular weight of Mn= 20969 g/mol, Mw= 48439 g/mol and a polydispersity of 2.310, see **Figure S4d**). Keeping all other conditions unchanged, polymer **P5** can be

prepared by replacing $TbCl_3 \cdot 6H_2O$ with $EuCl_3 \cdot 6H_2O$.

1.4 Characterizations

Fourier transform infrared (FT-IR) measurements were examined on the Bruker TENSOR 27 spectrometer in the range of 400−4000 cm^{-1} (16 scans, resolution: 4 cm^{-1}). Transmittance and UV-vis absorption spectra were by evaluated an Agilent Cary 100 UV-Vis spectrometer. Steady-state luminescence spectroscopy and lifetimes tests were obtained on Edinburgh Instruments FS920P spectrometer. Stress-strain curves were obtained on the CMT6104 with a stretch speed of 20 mm min^{-1}, the gauge length, width and thickness of the samples were about 10, 5 and 0.2 mm respectively, each test was conducted at least 5 times. For cyclic tensile tests, the maximum strain was set to be 100% (for different strains: 20%, 40%, 80%, 160%, 302%, 640%, 1000%, and 1500%) and appropriate consecutive cycles were conducted out. Differential scanning calorimetry (DSC) was evaluated on a TA DSC Q2000 differential scanning calorimeter in the range of −80 °C-200 °C with a heated or cooled rate of 10 °C/min in the atmosphere. The gel permeation chromatography (GPC) was recorded by Agilent PL-GPC50 and THF was taken as the mobile phase. Rheological performances measurements were obtained on an Anton Paar MCR-302 rheometer under ambient conditions. Frequency sweeps measurements were tested from 0.068 to 628 rad/s under 1% strain. Strain sweeps curve was tested with a strain of 0.001-1000% at 25 °C (frequency:1 Hz). Thermal gravimetric analysis (TG) was measured on America TA 209 SDT/Q600 instrument in the range of 25-800 °C under nitrogen atmosphere with a heating rate of 10 °C min^{-1}.

Polymer	PPG-NCO (g/mmol)	HDA (mg/mmol)	SP (mg)	Tpy-NH$_2$ (mg/mmol)	Tb / Eu (μL)
**P0**	1.15/0.5	46/0.4	0	0	0
**P1**	1.15/0.5	46/0.4	2	0	0
**P2**	1.15/0.5	46/0.4	2	15	225/0
**P3**	1.15/0.5	46/0.4	2	15	0/225
**P4**	1.15/0.5	46/0.4	0	15	225/0
**P5**	1.15/0.5	46/0.4	0	15	0/225

Table S1. The feed ratios of various polymers.

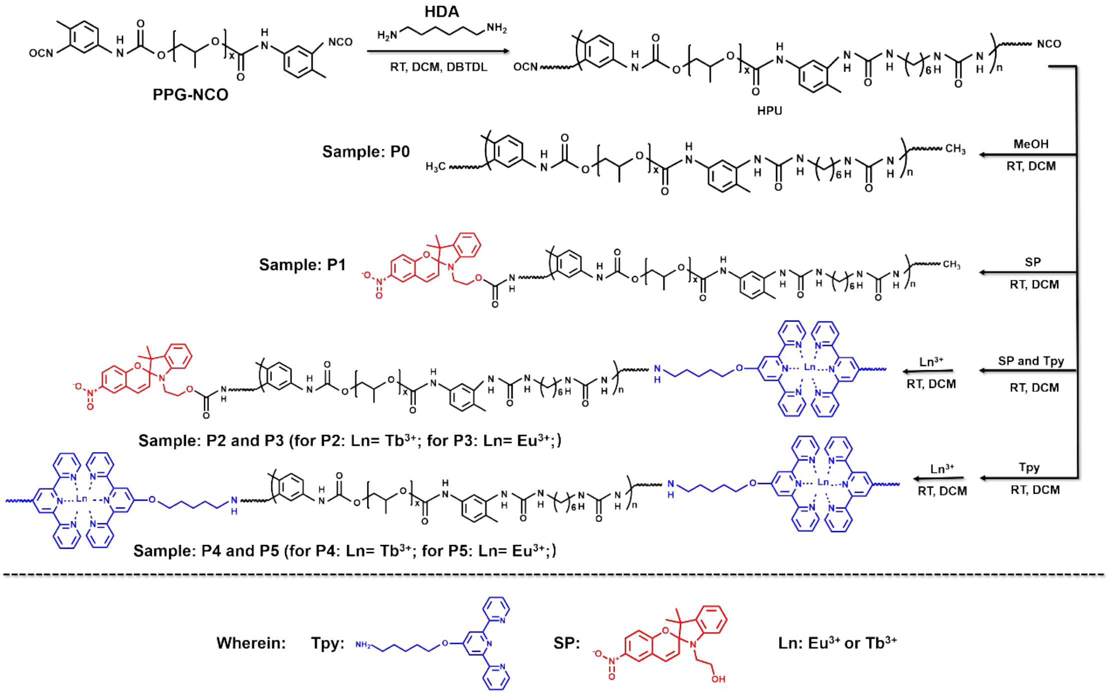

Figure S1. The synthesis routes of a series of polymers.

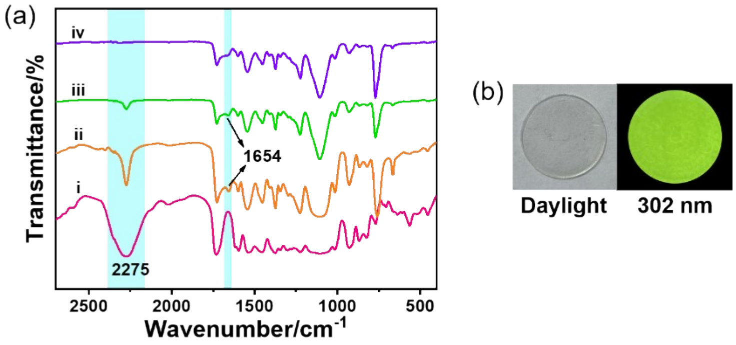

Figure S2. FT-IR spectra of a series of products, where i) **PPG-NCO**; 2) **HPU**; 3) **HPU-Tpy-SP**; iv) **P2**. (b) digital photos of **P2** under daylight and 302 UV light.

The successful preparation of intermediate and final materials can be demonstrated by their corresponding infrared (FT-IR) spectra. The FT-IR spectrum of **HPU** shows a weakened peak at 2275 cm^{-1} belongs to N=C=O together with the emergence of amide at 1654 cm^{-1} compared with that of PPG-NCO, demonstrating that the diisocyanate monomers had been converted to urea bonds.[1] ii) The intermediate product **HPU** obtained in step 1 was then reacted with Tpy-NH_2 and SP-OH to synthesize **HPU-Tpy-SP**. After that, the weakened peak of N=C=O at 2275 cm^{-1} was further decreased. iii) Finally, a proper amount of $LnCl_3{\cdot}6H_2O$ ethanol solution and excessive MeOH were introduce to the **PU-Tpy-SP** system, followed by the weakened N=C=O peak at 2275 cm $^{-1}$ completely disappears originated from the quenching effect of MeOH, which eliminated the residual isocyanate. Then, the solution was cast into PTFE to obtain transparent **P2** films**,** which can emit superior characteristic emission of the corresponding Tb^{3+}, implying the energy transfer from the Tpy to Tb^{3+} occurred, which demonstrates that the ligands coordinate with Tb^{3+} successfully (**Figure S3b)**.

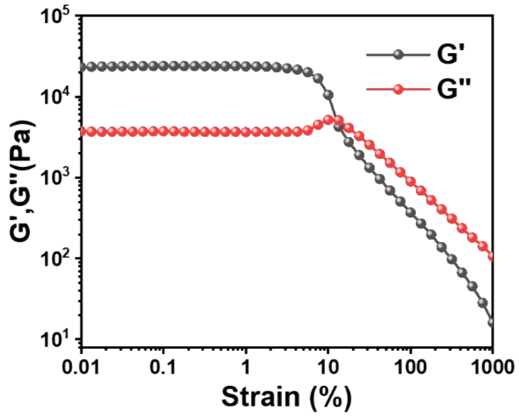

Figure S3. Strain sweep measurement of storage modulus (G′) and loss modulus (G″) at ω = 1 Hz with γ = 0.001-1000% of the resulting **P2** polymer.

Rheological curves show that the storage modulus (G′) of the photochromic polymer was higher than its loss modulus (G″) in the strain range of 0.001-13.4%, implying its solid-state behavior which is different from the raw material liquid PPG-NCO, thereby implying the successful formation of a typical cross-linked polymer network.

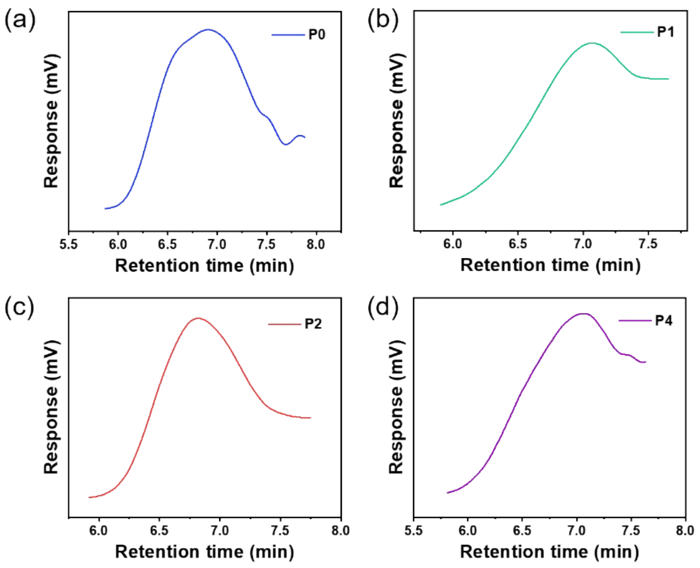

Figure S4. GPC curves of (a) **P0**; (b) **P1**; (c) **P2**; (d) **P4**.

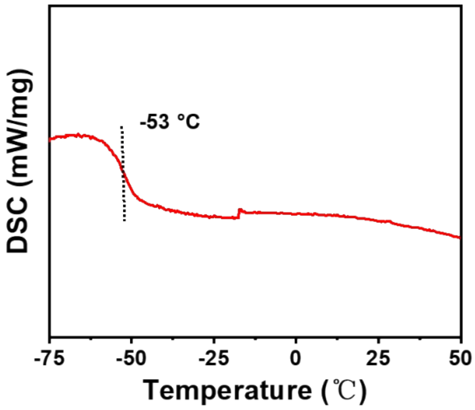

Figure S5. DSC curve of the representative sample **P2**.

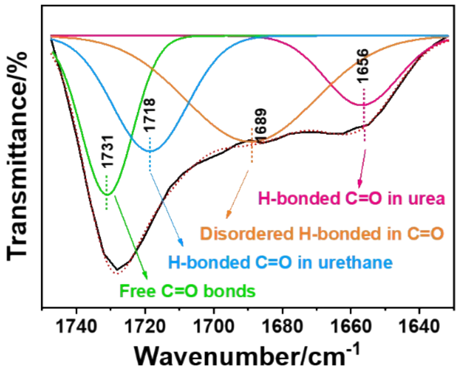

Figure S6. Transmittance spectra in the C=O stretching region (1747~1656 cm^{-1}) of the obtained **P2** material. The dashed lines are the fitted bands at 1731, 1719, 1689 and 1656 cm^{-1}.

These four peaks of **P2** in (1747~1656 cm^{-1}) belong to free C=O (1731 cm^{-1}, 22.8%), H-bonded in urethane C=O (1719 cm^{-1}, 24.2%), disordered H-bonded C=O in urea (1689 cm^{-1}, 38.7%) and ordered H-bonded C=O (1656 cm^{-1}, 14.3%), respectively.[2, 3]

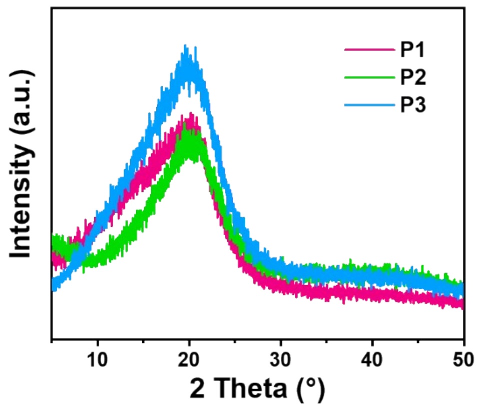

Figure S7. WAXD spectra of the obtained photosensitive polymers.

The wide-angle X-ray diffraction (WAXD) spectra of the obtained elastomers only show broadband absorption and without sharp XRD peaks, which are the representative WAXD patterns of amorphous polymers.

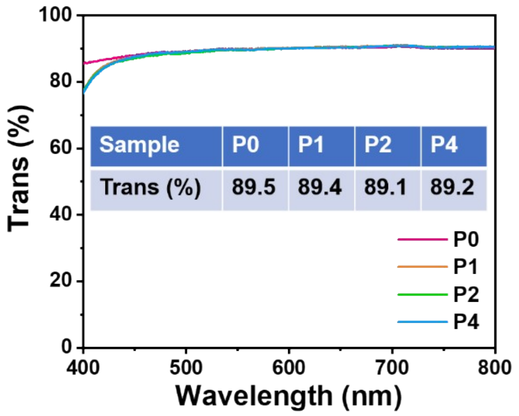

Figure S8. Transmittance spectra of **P0**, **P1**, **P2**, and **P4**.

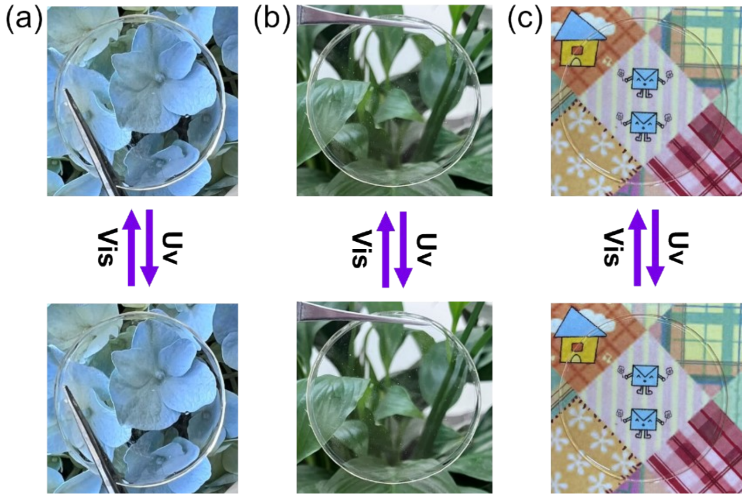

Figure S9. Sample (a) **P0**, (b) **P4** and (b) **P5** before and after being UV light treatment.

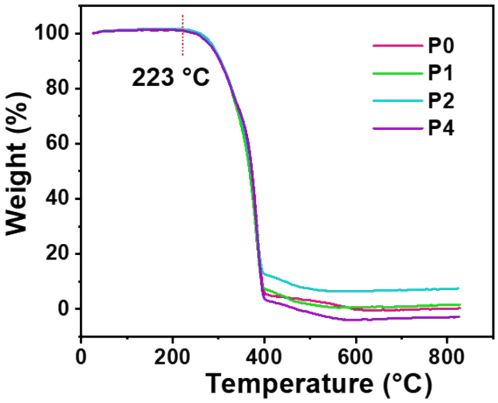

Figure S10. TG curve of the representative sample **P2**.

Sample	Young's Modulus (MPa)	Tensile strength (MPa)	Elongation at break (%)	Toughness (MJ m^{-3})
**P1**	11.75±0.32	14.48±0.39	2643±72	214.11±15.39
**P2**	13.63±0.40	20.19±0.86	2354±48	229.79±12.65
**P3**	12.84±0.28	19.09±0.13	2258±53	204.49±18.91
**P4**	13.53±0.45	21.06±0.18	2409±67	249.70±16.87
**P5**	13.12±0.32	21.06±0.86	2273±44	217.41±15.84

Table S2. Summary of mechanical properties of a range of materials (stretch speed: 50 mm min^{-1}).

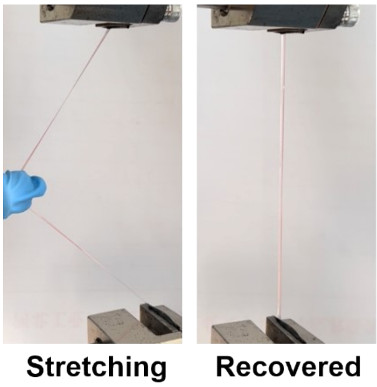

Figure S11. Fast-recovery behavior of sample **P2**.

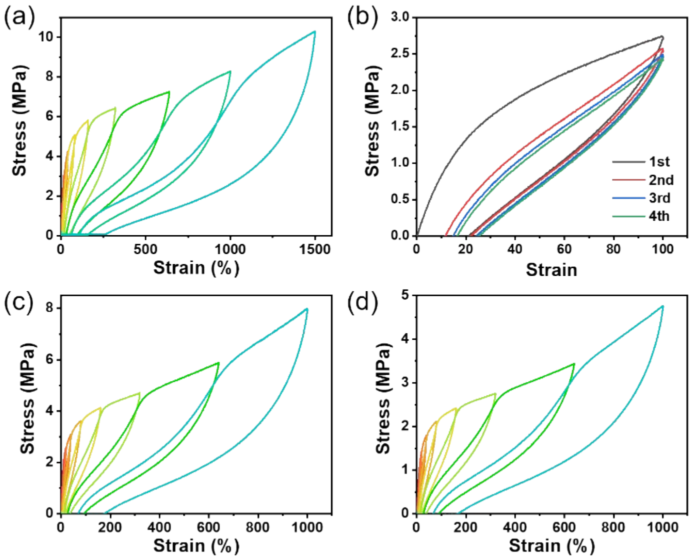

Figure S12. **P2** was loaded and unloaded to (a) different strains and (b) 100% strain. (c) **P4** and (d) **P1** were loaded and unloaded to different strains.

Sample	1st	2nd	3rd	4th
**P2**	1019.8	430.0	371.4	343.5

Table S3. Energy dissipation (KJ m^{-3}) in continuous cyclic loading and unloading of the representative **P2** sample.

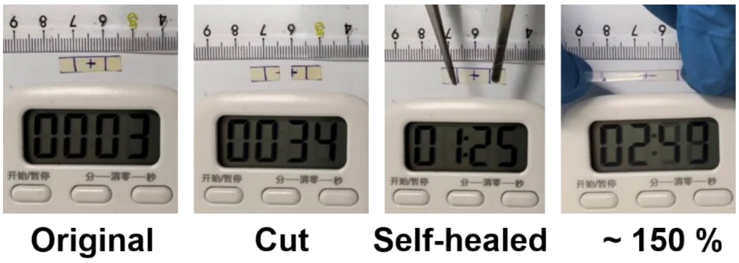

Figure S13. (a) Self-healing performance photos of fractured photosensitive **P2**.

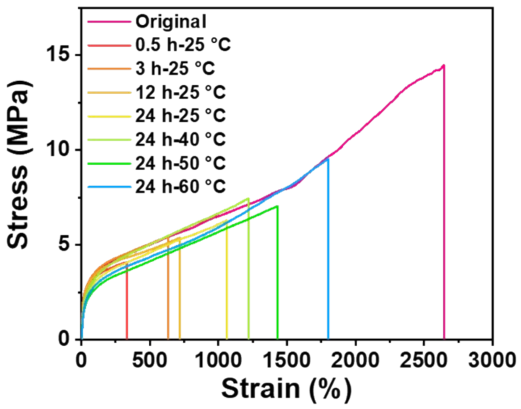

Figure S14. Stress-strain curves of fractured photosensitive **P1** samples self-healed for different times at room temperature (RT) and for 24 h at different temperatures (stretch speed: 50 mm min^{-1}).

Time/Temperature	Stain (%)	Stress (MPa)	Toughness ($MJ\ m^{-3}$)	η (%)
0.5 h/RT	253±15	4.53±0.32	8.97±1.52	3.9±0.66
3 h/RT	467±23	4.76±0.41	17.35±2.16	7.6±0.94
12 h/RT	811±28	6.47±0.40	38.38±4.25	16.7±1.85
24 h/RT	1144±31	7.49±0.53	58.33±4.69	25.4±2.05
24 h/40 °C	1246±18	10.12±0.45	76.52±8.5	33.30±3.71
24 h/50 °C	1521±21	11.92±0.26	105.59±9.3	45.95±4.06
24 h/60 °C	1842±33	14.92±0.43	140.25±10.3	61.03±4.49

Table S4. Summary of self-healing efficiency of **P2** self-healed for different times at room temperature (RT) and for 24 h at different temperatures.

Time/Temperature	Stain (%)	Stress (MPa)	Toughness (MJ m^{-3})	η (%)
0.5 h/RT	333±10	4.07±0.18	11.42±0.50	5.33±0.23
3 h/RT	630±18	5.44±0.06	27.29±0.21	12.75±0.09
12 h/RT	718±23	5.37±0.32	30.43±3.82	14.21±1.79
24 h/RT	1059±36	6.29±0.68	48.39±3.24	22.61±1.51
24 h/40 °C	1217±31	7.45±0.34	64.03±5.36	29.92±2.50
24 h/50 °C	1430±25	7.91±0.28	71.32±6.20	33.32±2.89
24 h/60 °C	1798±37	9.54±0.47	103.42±5.44	48.32±2.54

Table S5. Summary of self-healing efficiency of **P1** self-healed for different times at RT and for 24 h at different temperatures.

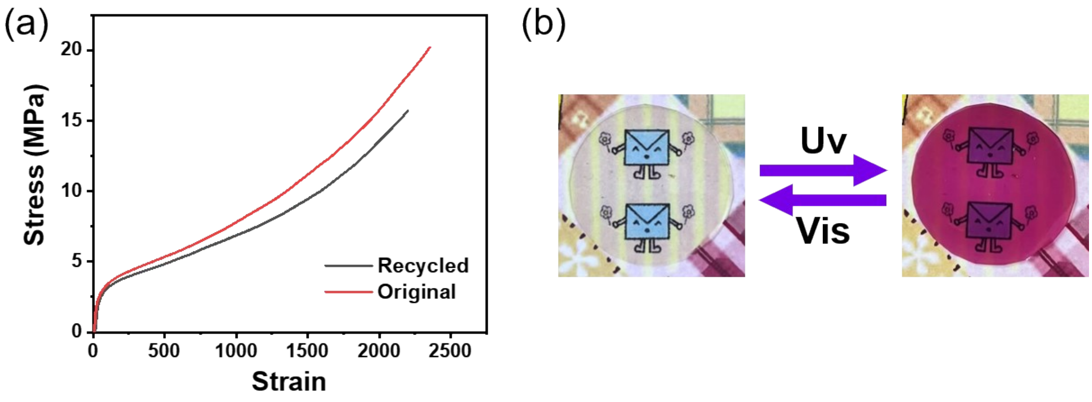

Figure S15. (a) Stress-strain curves of original and recycled **P2** samples. (b) Photochromic ability of the recycled sample.

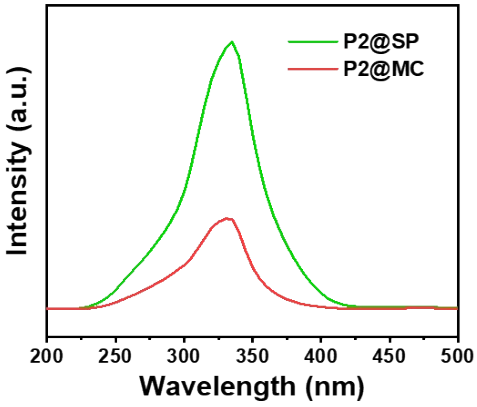

Figure S16. Excitation curves of **P2** at both SP-state and MC-state monitored at 545 nm.

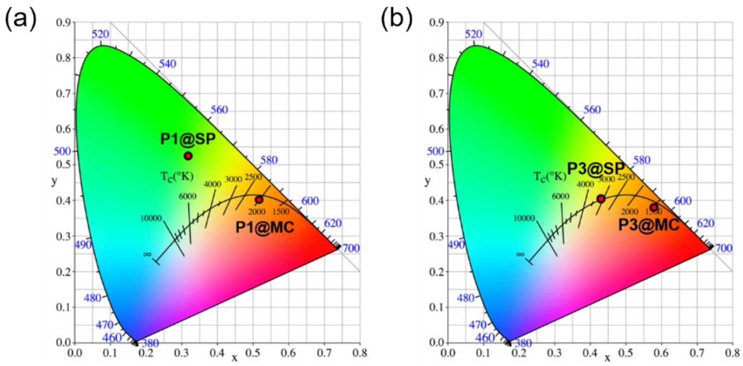

Figure S17. Emission colors of (a) **P1** and (b) **P3** at both SP-state and MC-state in the CIE 1931 diagrams (Ex: 325 nm).

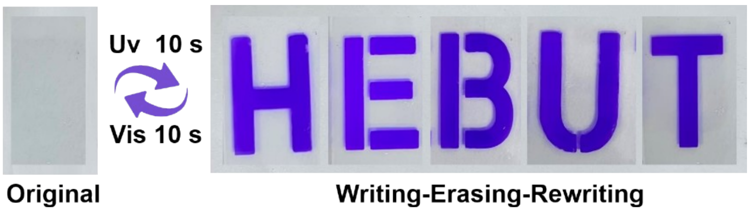

Figure S18. (a) UV-induced writing / rewriting and Vis-induced erasing behavior on a **P1** film (about 3 cm × 4 cm).

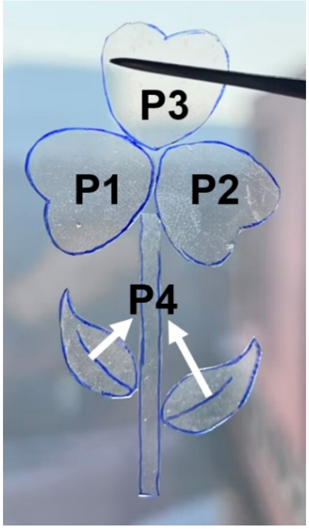

Figure S19. The distribution of various samples on the pattern.

References

[1] D.-P. Wang, Z.-H. Zhao, C.-H. Li, Universal self-healing poly (dimethylsiloxane) polymer crosslinked predominantly by physical entanglements, ACS Applied Materials & Interfaces 13(26) (2021) 31129-31139.

[2] J. Cao, C. Lu, J. Zhuang, M. Liu, X. Zhang, Y. Yu, Q. Tao, Multiple hydrogen bonding enables the self-healing of sensors for human–machine interactions, Angewandte Chemie 129(30) (2017) 8921-8926.

[3] Y. Song, Y. Liu, T. Qi, G.L. Li, Towards dynamic but supertough healable polymers through biomimetic hierarchical hydrogen-bonding interactions, Angewandte Chemie International Edition 57(42) (2018) 13838-13842.
